# A two-site survey of medical center personnel’s willingness to share clinical data for research: implications for reproducible health NLP research

**DOI:** 10.1186/s12911-019-0778-z

**Published:** 2019-04-04

**Authors:** Chunhua Weng, Carol Friedman, Casey A. Rommel, John F. Hurdle

**Affiliations:** 1 Department of Biomedical Informatics, Columbia University, New York City, NY 10025 USA; 2 Department of Biomedical Informatics, University of Utah, Salt Lake City, UT 84108 USA

## Abstract

**Background:**

A shareable repository of clinical notes is critical for advancing natural language processing (NLP) research, and therefore a goal of many NLP researchers is to create a shareable repository of clinical notes, that has breadth (from multiple institutions) as well as depth (as much individual data as possible).

**Methods:**

We aimed to assess the degree to which individuals would be willing to contribute their health data to such a repository. A compact e-survey probed willingness to share demographic and clinical data categories. Participants were faculty, staff, and students in two geographically diverse major medical centers (Utah and New York). Such a sample could be expected to respond like a typical potential participant from the general public who is given complete and fully informed consent about the pros and cons of participating in a research study.

**Results:**

Two thousand one hundred forty respondents completed the surveys. 56% of respondents were “somewhat/definitely willing” to share clinical data *with* identifiers, while 89% of respondents were “somewhat (17%)/definitely willing (72%)” to share *without* identifiers. Results were consistent across gender, age, and education, but there were some differences by geographical region. Individuals were most reluctant (50–74%) sharing mental health, substance abuse, and domestic violence data.

**Conclusions:**

We conclude that a substantial fraction of potential patient participants, once educated about risks and benefits, would be willing to donate de-identified clinical data to a shared research repository. A slight majority even would be willing to share absent de-identification, suggesting that perceptions about data misuse are not a major concern. Such a repository of clinical notes should be invaluable for clinical NLP research and advancement.

## Background

Reproducibility of Natural Language Processing (NLP) methods and comparison of results is the cornerstone of biomedical NLP research, but this requires that patient data including clinical textual notes be made shareable. This is a challenge owing to confidentiality and privacy issues. Therefore, a goal of many NLP researchers is to create a shareable repository of clinical notes that has breadth (from multiple institutions) as well as depth (as much longitudinal data as possible from individuals). Corn noted the need to preserve the “clinical phenome,” which inspired much of our work here [1]. A corpus of clinical notes would enable robust testing of clinical NLP tools created at different institutions, something extremely difficult to do under the widely acknowledged constraints [2, 3] imposed on cross-institutional research by the Health Insurance Portability and Accountability Act (HIPAA).

The de facto norm for sharing clinical notes is to apply de-identification software to remove as much protected health information (PHI) as possible and then seek local Institutional Review Board (IRB) approval to share the narratives under a waiver of patient consent and authorization. The distribution of such a corpus is always managed under a data sharing agreement. Some successful examples include the MIMIC II database from Beth Israel Deaconess Hospital [4] (intensive care unit notes); the Cincinnati Children’s Hospital corpus used for mapping text to ICD-9 codes [5] (radiology reports); and the University of Pittsburgh’s BLULab corpus (a mix of note types, but unfortunately no longer available).

While state-of-the-art of de-identification software is quite accurate [6–8], it is far from perfect. In 2015 Stubbs et al. published a review of the state-of-the-art in clinical note de-identification showing that 3 of the 10 best systems achieved F1 scores over .90 and 7 of the top 10 scored over .75 [9]. This performance is similar to manual scrubbing of PHI [10]. Dorr et al. found the typical note contained, on average about eight instances of PHI. Therefore in 100 notes, a state-of-the-art de-identification system could be missing 40 or more instances of PHI [10]. In light of that kind of performance the University of Utah and Columbia University IRBs do not sanction the release of de-identified clinical notes under waivers of patient consent and authorization.

An alternative, and more ethical, approach is to seek the permission of patients in order to share their data. We are unaware of any shareable corpus of clinical notes that relies on the informed consent and authorization of patients. This is unfortunate because the typical IRB is much more comfortable evaluating risk and benefit under a consent model than under the Safe Harbor De-Identification or the Statistical Certification models permitted under HIPAA. Malin et al. provide technical and policy summary of these models [6]. Seeking a patient’s permission to share his or her clinical notes for research purposes effectively eliminates institutional risk under HIPAA and Health Information Technology for Economic and Clinical Health Act (HITECH). But it cannot be assumed that a significant number of potential participants would be willing to consent to allowing researchers to share the highly personal information found in clinical notes. This is the research question we explore in this paper.

### Prior work

The literature is mixed on the question of how willing patients are to share their medical data. Much of the recent literature in this area focuses on the sharing of genetic/genomic data, which carry unique ethical issues. When a person shares their own whole genome sequence they are also sharing information about their parents, siblings, children, and other relatives. Given the complexities of these ethical issues, we restrict ourselves here to a set of five representative studies of patients’ willingness to share non-genetic clinical data.

Weitzman et al. probed 261 users about their willingness to share a wide variety of personal medical data with both “outside providers” (clinicians) as well as with public-health decision-makers at the state/local public health department [11]. Users were young adults and parents who use a personally controlled health record (i.e., an electronic health record [EHR] that contains detailed medical information and that allows a patient to select “who can see what”). They found that more users were willing to share all categories of information with the state/local public health authority (63.3%) than with an outside provider (54.1%). They also found that only a small number of users would not share any category of information (outside provider 5.2% or public health authority 7.9%). In a separate study of 151 early adopters of the personally controlled health record who were asked about sharing personal data for health research generally, Weitzman et al. found that 91% were willing to share medical information [12].

Zulman et al. surveyed a convenience sample of veterans (*n* = 18,471) using the Veteran Administration’s (VA) MyHealtheVet personal health record and found 78.7% would share *some* information with some categories of individuals such as family members, friends, neighbors, and non-VA health care providers [13]. Willingness to share varied by the category of the data recipient, ranging from 2% willing (sharing with friends) to 25% willing (non-VA care providers) to a maximum of 62% willing (spouse/partner). The survey did not query about sharing for research purposes, but we mention the study because of its impressive sample size and because it illustrates that patients are selective about whom they wish to share their data with.

The California Health Care Foundation (CHCF) conducted two surveys of the public’s attitudes about medical data privacy, the most recent in 2005 [14]. That survey sampled 1000 adults nationally with an additional 1000 in the state of California. It found that 67% of respondents were “somewhat concerned” or “very concerned” about the privacy of their medical records. Interestingly, 12.5% of respondents reported intentionally under-utilizing the healthcare system out of privacy concerns. Conversely, when asked if respondents felt that “researchers have the right to use” personal medical data to improve healthcare, 42% answered in the affirmative.

Grande et al. published a study designed to detect differences in the public’s attitude about sharing clinical data, including genetic/genomic data, for specific purposes [14]. They found that using data for marketing or other commercial purposes were negatively correlated with a willingness-to-share, but that participants were more favorably disposed to sharing for research. They also found that Hispanics and African Americans were less differentiating than Caucasians about data use. A limitation of this study was the complexity of the survey instrument and its use of direct compensation (e.g., participants in the survey network were invited to join four to six surveys per month and were paid for their time).

All five of these reports illustrate that patients are thoughtful about medical privacy issues and are selective about who should see or access their personal medical data. The two Weitzman studies and the CHCF study, however, present striking differences in the public’s attitude about sharing medical data for research purposes. Willingness-to-share varied from 42% [15] to 63.3% [11] to 91% [12]. Clearly there is room for continued research in this important and timely area, which provides the rationale for the study presented below.

### Objective

We set out to investigate the degree to which well-informed individuals might be willing to share clinical information and to characterize the degree to which individuals would share specific clinical content (e.g., demographic data versus reproductive health data). We were also interested in what respondents thought about *others* willingness to share and what respondents thought about sharing medical data about deceased family members. Huser and Cimino make a persuasive case for the utility of the latter, explaining in a recent publication the regulatory advantages of using deceased individuals’ EHR data [16].

We sampled faculty, students, and staff at major medical centers under the premise that they are the most likely to understand both the risks and the benefits of participating in research, a point we expand on in the Discussion section. That is, they could be expected to respond like a typical potential participant from the general public who is given complete and fully informed consent.

## Methods

The survey was designed and administered initially at the University of Utah (UU, in the Intermountain West) using an email-based survey distribution system. Participation was high, encouraging us to add another medical center in a different geographical location: Columbia University (CU, in the Northeast), which also used an email-based survey distribution system similar to UU’s.

### Survey design

The survey had four short sections. All questions were designated as optional, so the data set contains occasional null values. The demographics section consisted of the four questions shown in Table 1. We decided against collecting racial/ethnicity information. In 2007 the Department of Education issued guidance [17] for properly collecting such data and we felt that its complexity diminished the simplicity we were striving for in our survey. In hindsight, we would have added the six pre-2007 federal race/ethnicity categories.

**Table 1:** Demographic questions

What is your gender?	• Male • Female
What is your age?	• 18–25 • 26–40 • 41–55 • 56 or older
What is the highest level of education you have completed?	• Less than high school • High school/GED • Some college • Bachelor’s degree or college graduate • Graduate or professional degree
What is your employment category? (Choose the one that you identify with the most)^a^	• Staff • Faculty • Student

^a^CU desired more detail about employment categories, listing seven in their survey. These were mapped to the three shown here when we performed our data analysis

The “willingness to share” section consisted of four questions based on a Likert scale. It is shown in Table 2. We chose the order of the questions intentionally. The idea was to move the respondent from thinking more broadly about other people to thinking more specifically about themselves. The difference between the third and fourth questions centers on whether respondents’ attitudes changed if the data were de-identified or not.

**Table 2:** Willingness to share questions

**•** No, not all • Somewhat unwilling • Might be willing • Somewhat willing • Yes, definitely	
1) Do you think MOST people would be willing to share their medical data/records with qualified scientists for medical research purposes?	
2) For deceased FAMILY members, would you be willing to donate their medical records/data to qualified scientists for medical research purposes?	
3) Would you be willing to share some or all of YOUR medical data/records with qualified scientists for medical research purposes, even if it contained identifying information like names and addresses?	
4) Would you be more likely to share some or all of YOUR medical data/records if your name, address, and other identifying information have been removed first?	

The third section, “willingness to share by data category,” presented 15 broad data categories and is shown in Table 3. We chose the categories starting with obvious choices such as demographics and vital signs and augmented those with categories loosely aggregated from the National Committee on Vital and Health Statistics privacy report [18]. Again, we chose the order of the questions intentionally. With the exception of “mental health,” respondents were asked to consider the most “innocuous” data first and progressed to increasingly more sensitive data. Mental health was inserted in the middle because we especially wanted to capture that data point.

**Table 3:** Willingness to share by data type questions

• No, not all • Somewhat unwilling • Might be willing • Somewhat willing • Yes, definitely		
1) Demographics (like age, gender, ethnicity)	2) Vitals (blood pressure, weight, height)	3) Medications
4) Lab test results (blood, urine)	5) Diagnostic reports (x-rays, mammograms)	6) Disabilities
7) Mental Health	8) Childhood Diseases	9) Surgeries
10) Chronic Illnesses (diabetes, hypertension)	11) Cancer	12) Reproductive Health
13) Domestic Violence	14) Alcohol & tobacco use	15) Substance Abuse

The final section, shown in Table 4, asked a question designed to probe why a respondent might be unwilling to share any data at all. These were based on the concerns raised by participants in the CHCF survey mentioned above [15]. The survey also provided respondents a chance to express the same concerns in free text.

**Table 4:** Reasons why respondents are unwilling to share

If you are unwilling to share *any* clinical information for research purposes, please indicate why (check as many as you want).	
▪ Not applicable, I am willing to share this information.	
▪ It would make me uncomfortable to share this information.	
▪ I am afraid my information will be used by the government.	
▪ I don’t trust that my information will be kept confidential.	
▪ It may compromise my future health care or insurance.	

### Survey administration

After obtaining local IRB approval, both sites administered the survey electronically through an email distribution system. CU also used the Mechanical Turk crowd-sourcing Internet marketplace^19^ to mount a separate experiment focused on the general public. That work will be described in a separate publication. In addition to creating a compact survey to increase participation, each site also offered an incentive where participants could opt-in to a drawing for a prize. UU offered one $250 shopping card and two $100 shopping cards. CU offered one Amazon Kindle with a value of $150. The data from the UU survey was returned to the study team in the form of a comma-delimited file. CU’s IRB would not allow UU to host CU’s participant data, so the CU team provided data in Excel format. When a survey closed, any identifying information collected from respondents who chose to opt-in to drawings was separated from response data. As soon as a winner was selected (at random) all identifying data were deleted. The response data were forwarded to UU for consolidation. All statistics cited in this paper were carried out using Stata, version 12, College Station, Texas.

## Results

A total of 2140 respondents completed the surveys (CU 449 and UU 1691). The demographic characteristics of the combined sample are shown in Table 5. That same table shows how willing participants were to share personal data with and without identifying information, broken down by demography. It also shows how each demographic subset felt about sharing deceased family member data. Pearson’s chi-square shows that there was no significant within-demographic variation. Although some would argue the Likert scale we used is not sufficiently continuous (or interval-scaled) to permit a chi-square analysis, investigations by simulation studies have found there is no harm in treating such a scale as a continuous variable [19].

**Table 5:** Characteristics of the sample and willingness to share own and decesased family data

Participant characteristics	N (percentage)	Willing or somewhat willing to share *own* data, *identified*	Willing or somewhat willing to share *own* data, *de-identified*	Willing or somewhat willing to share *deceased family member* data
Age				
18–25	189 (6.8%)	54.1%	90.0%	80.4%
26–40	1195 (43.3%)	51.3%	89.6%	81.5%
41–55	929 (33.6%)	51.7%	88.3%	80.9%
56+	450 (16.3%)	55.5%	90.9%	81.19%
Pearson *chi-square*		4.07, *p* = 0.25 (slight differences due to chance)	3.0, *p* = 0.41 (slight differences due to chance)	2.47, *p* = 0.48 (slight differences due to chance)
Gender				
Male	768 (27.8%)	52.3%	89.7%	82.3%
Female	1992 (72.2%)	52.6%	89.1%	81.4%
Pearson *chi-square*		0.02, *p* = 0.88 (slight differences due to chance)	0.18, *p* = 0.67 (slight differences due to chance)	0.25, *p* = 0.62 (slight differences due to chance)
Education				
High school/GED	74 (2.7%)	59.5%	85.1%	83.8%
Some college	512 (18.5%)	53.5%	89.4%	81.0%
College graduate	927 (33.5%)	52.2%	89.1%	80.6%
Graduate/Prof. degree	1251 (45.3%)	51.9%	89.5%	82.7%
Pearson *chi-square*		1.88, *p* = 0.6 (slight differences due to chance)	1.44, *p* = 0.70 (slight differences due to chance)	2.02, *p* = 0.57 (slight differences due to chance)
Employment status				
Faculty	613 (22.2%)	51.9%	88.4%	81.7%
Staff	1907 (69.0%)	52.2%	89.6%	82.2%
Student	140 (5.1%)	61.3%	92.1%	80.7%
Other	102 (3.7%)	52.0%	85.3%	73.5%
Pearson *chi-square*		4.67, *p* = 0.2 (slight differences due to chance)	3.57, *p* = 0.31 (slight differences due to chance)	4.98, *p* = 0.17 (slight differences due to chance)

The participants were remarkably consistent across demographics subsets: on average 53% of respondents were somewhat (34%) or definitely willing (19%) to share clinical data with identifiers, while 89% of respondents were somewhat (18%) or definitely willing (71%) to share without identifiers. There were subtle differences, though, when participants were asked about the category of data they would be willing to share. As Table 6 shows, Mental Health, Domestic Violence, and Substance Abuse data were of the most concern to respondents, while Disability, Chronic Illness, Reproductive History, and Alcohol/Tobacco-Use data caused some concern.

**Table 6:**
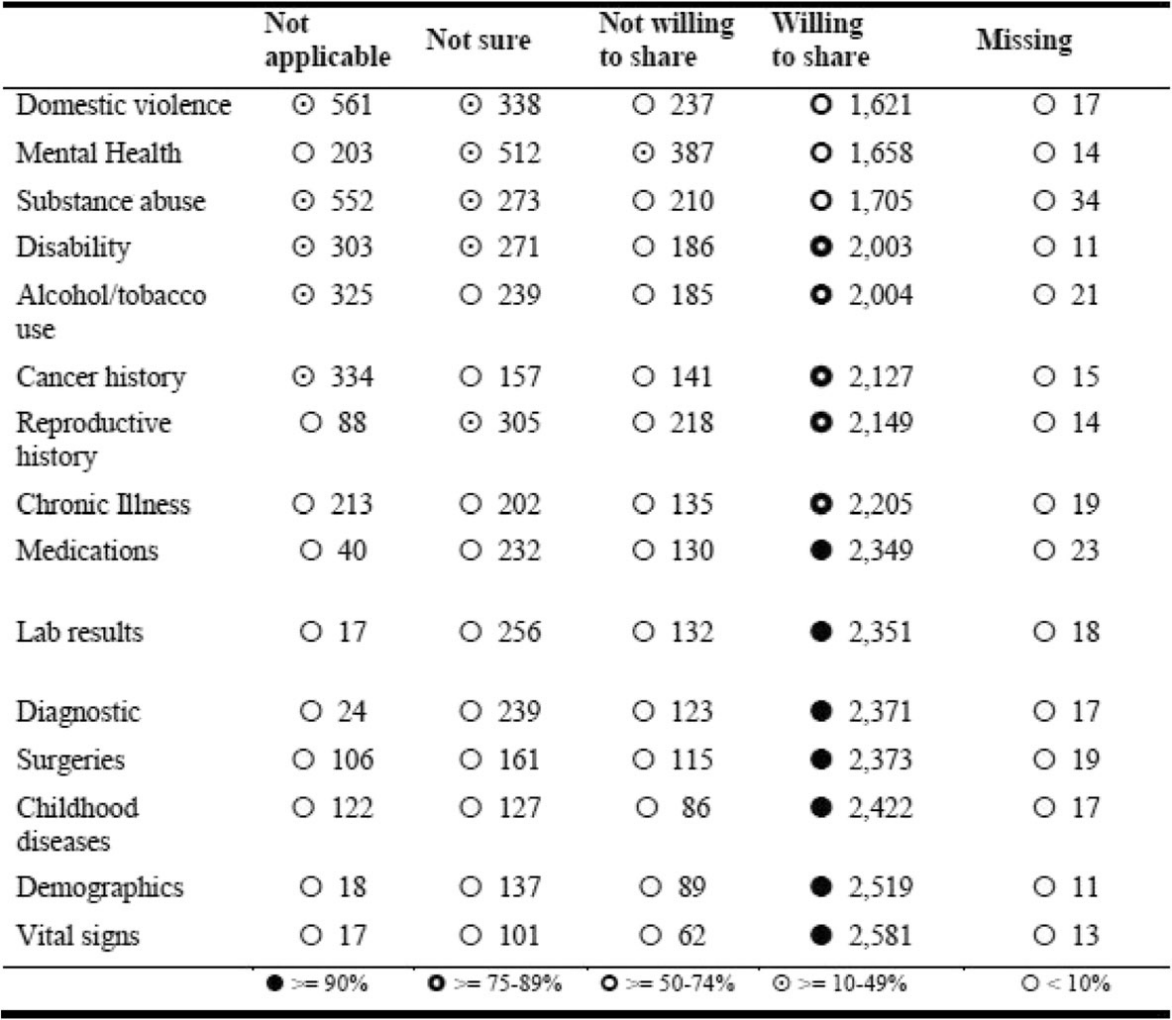
Distribution of willingness to share own data by data category, *sorted from least to most willing to share*

We analyzed the demographic breakdown of data sharing concerns, as shown in Table 7. In general, males were more willing to share data of any kind, and older participants were less likely to be willing to share, but note that Table 7 shows a large degree of variability and no systematic demographic trends are clear from these data. That said, younger participants seemed less concerned about alcohol, tobacco, and substance abuse data.

**Table 7:** Significant differences in willingess to share own data by respondent demographics (*p* < = 0.05), sorted from least to most variation in willingness to share

Substance abuse	Female↓; Male↑; High school and Some college and College degree↑; Grad./Prof degree↓; 26–40↑; 41–56 and 56 + ↓
Domestic violence	Female↑; Male↑; Some College and College degree↑; Grad./Prof degree↓; Staff↑; Other and Faculty↓
Mental Health	Some College↑; College Graduate↑; Grad./Prof degree↓; Faculty↓; Staff↑
Disability	Male↑; Female↓; Grad./Prof degree↓; Some College↑; Faculty↓; Staff↑
Lab results	College degree↑; Grad./Prof degree↓; Faculty↓; Staff ↑
Cancer history	Female↓; Male↑; 26–40↑; 41–56 and 56 + ↓
Reproductive history	Female↑; Male↓; 26–40↑; 41–56 and 56 + ↓
Diagnostic	Faculty↓; Staff↑; Grad. Prof degree↓
Childhood diseases	18–25↓; 56 + ↑; Faculty↓; Student↓
Chronic Illness	26–4↑; 41–55 and 56 + ↓
Demographics	Male↑; Female↓
Alcohol/tobacco use	26–40↑; 41–56↓
Vital signs	None
Medications	None
Surgeries	None

↑ = more willing to share; ↓ = less willing to share

It is interesting to note that when asked about a respondent’s sense of “Do you think MOST people would be willing to share?” there was no significant difference by education or employment type, but there was a significant difference between how men and women viewed others’ willingness to share: women viewed others as being more conservative (expected *n* = 1046, actual *n* = 1014) than did men (expected *n* = 403, actual *n* = 435; Pearson chi-square 7.483, *p* = 0.006). There was also a significant difference by age group, most notably in the age 26–40 group who thought people would be more conservative generally (expected *n* = 627, actual *n* = 608). Yet when asked about their own willingness to share, this age group was consistently more likely to share. Contrarily, the 56+ age group thought people would be less conservative generally (expected *n* = 236, actual *n* = 261; Pearson chi-square 7.901, *p* = 0.048) but they themselves were typically more conservative, as shown in Table 7.

There were notable differences in the means, as measured by the Kruskal-Wallis chi-square test, by geographic region. There was no significant difference across the five willingness-to-share categories between the Northeast and the Northwest (willingness-to-share with identifiers: Kruskal-Wallis chi-square = 0.78, *p* = 0.38; willingness-to-share without identifiers: Kruskal-Wallis chi-square = 1.85, *p* = 0.17). However, there was a significant difference between the Intermountain West and the Northwest (willingness-to-share with identifiers: Kruskal-Wallis chi-square = 28.64, p = 0. 001; willingness-to-share without identifiers: Kruskal-Wallis chi-square = 3.35, *p* = 0.067) and the Intermountain West and the Northeast (willingness to share with identifiers: Kruskal-Wallis chi-square = 32.05, *p* = 0.000; willingness to share without identifiers: Kruskal-Wallis chi-square = 10.51, *p* = 0.001). CU respondents reported being more “somewhat willing to share” than UU, while those from UU reported more “Yes, definitely” more often. UU respondents, even after adjusting for their higher representation in the sample, were more definitive about their willingness to share their own data.

## Discussion

We set out to answer the question “Is it reasonable to expect that a significant number of well-informed potential participants would be willing to allow researchers to share the highly personal information found in a medical chart?” The answer is a “qualified yes” for our cohort, especially if efforts are made to de-identify the data. This finding holds across major demographic groups, but there are some categories of data that are more sensitive than others (i.e., Mental Health, Domestic Violence, and Substance Abuse data). Regional differences, though minor, were evident.

Our decision to focus on members of major medical center communities has both limitations and strengths. These participants are in an excellent position to understand both the compelling need for individuals to enroll in clinical research as well as the potential adverse outcomes that could follow the disclosure of personally identifying health data. We view this cohort as a proxy for typical potential participants after being educated by an informed consent process. That is, our cohort offers insight into the public’s willingness to share after understanding the pros and cons of participation. The downside of using this sample is the obvious potential for a systematic bias resulting from the lack of true randomization.

Although we lack the space here to present them in detail, we did collect data from the same survey offered to a sample of the general public through Amazon’s Mechanical Turk service [20]. We had 1764 respondents (58% male, 83% aged 18–40; 89% some college or college graduate). On average 40% of respondents were somewhat (25%) or definitely willing (15%) to share clinical data *with* identifiers (overall 13 percentage points less than the medical center population), while 74% of respondents were somewhat (23%) or definitely willing (51%) to share *without* identifiers (overall 15 percentage points less than the medical center population). The general public sample was more reticent than the medical center population to share, but the sample displayed a strong willingness to share deidentified data nonetheless. We speculate that the absence of a process like informed consent, or the absence of insight into medical research, contributed to the Amazon sample’s lower willingness to share.

The medical centers samples’ gender imbalance is puzzling. UU completed its survey first and used grocery-shopping cards as an incentive. We considered that this incentive might be more appealing to women, so CU used the more gender-neutral incentive of tablet devices. This did not change the gender imbalance, however. Since willingness-to-share did not change across any demographic group, including gender, we do not consider this limitation to be significant. Note that the nature of the campus email survey systems we used precludes establishing a proper response rate because we could not determine denominators of “all recipients.” That said, the sample size remains credible. In relation to other surveys that similarly probe the public’s willingness to share clinical data, our sample size is larger than all but a few. We probed willingness-to-share by data category, which is novel. To the best of our knowledge, this is the first survey to assess attitudes about both identifiable and de-identified data sharing. Our findings are consistent with the many related studies: patients are generally willing to allow their private data used in research, and they are thoughtful about that use. Finally, the study is limited to two geographical regions. It would informative to replicate the study in other areas.

The final question of the survey probed reasons why unwilling participants may not want to share their medical records. We collected optional free-text comments from both the medical center and the Amazon samples. We plan a thorough topic analysis of those comments to better understand participant reluctance. In brief, about 25% of the medical center population answering this question (overall *n* = 1449) cited concerns about maintaining confidentiality and an equal number cited concerns about compromised health insurance. Fewer than 10% cited data misuse by the government as a reason.

Our long-term goal is to build a shareable note repository using fully informed consent explicitly to avoid the constraints imposed by HIPAA and the HITECH. We envision a best-effort de-identification process followed by an interactive consent process where participants are allowed to redact note types that they feel uncomfortable sharing. The exact nature of this interaction will be the subject of future work, but it will be critical to detail the potential risks inherent in malicious re-identification. The data collected here offers insights into the categories of data we should be especially careful to highlight in that process.

## Conclusion

We conclude that a substantial fraction of potential patient participants, once educated about risks and benefits, would be willing to donate de-identified clinical data to a shared research repository. A slight majority even would be willing to share absent de-identification, suggesting that perceptions about data misuse are not a major concern. Such a repository of clinical notes should be invaluable for clinical NLP research and advancement.

## Abbreviations

CHCF: California Health Care Foundation
CU: Columbia University
EHR: Electronic health record
HIPAA: Health Insurance Portabilty and Accountability Act
HITECH: Health Information Technology for Economic and Clinical Health Act
IRB: Institutional Review Board
NLP: Natural language processing
PHI: Protected health information
UU: University of Utah
VA: Veterans Administration (a Department of the U.S. federal government)
